# Four-dimensional computed tomography as first-line imaging in primary hyperparathyroidism, a retrospective comparison to conventional imaging in a predominantly single adenoma population

**DOI:** 10.1186/s41824-024-00198-5

**Published:** 2024-05-01

**Authors:** Jorian P. Krol, Frank B.M. Joosten, Hans de Boer, Marie Louise E. Bernsen, Cornelis H. Slump, Wim J.G. Oyen

**Affiliations:** 1 Department of Radiology & Nuclear Medicine, Rijnstate Hospital, Wagnerlaan 55, Arnhem, 6815AD The Netherlands; 2 Department of Robotics and Mechatronics, Faculty of Electrical Engineering, Mathematics and Computer Sciences, University of Twente, Enschede, The Netherlands; 3 Department of Internal Medicine, Rijnstate Hospital, Arnhem, The Netherlands; 4 Department of Biomedical Sciences and Humanitas Clinical and Research Centre, Department of Nuclear Medicine, Humanitas University, Milan, Italy; 5 Department of Radiology & Nuclear Medicine, Radboud University Medical Centre, Nijmegen, The Netherlands

**Keywords:** Primary hyperparathyroidism, Four dimensional computed tomography, Ultrasound, [^99m^Tc]Tc-Sestamibi SPECT, [^18^F]fluorocholine PET/CT

## Abstract

**Background:**

To determine the use of four-dimensional CT as first-line imaging compared to the traditional combination of ultrasound and [^99m^Tc]Tc-Sestamibi SPECT.

**Materials and methods:**

Retrospective review of preoperative imaging in patients with primary hyperparathyroidism, who underwent parathyroidectomy between 2012 and 2021. In one group, the combination ultrasound and [^99m^Tc]Tc-Sestamibi SPECT was used as first-line imaging (*n* = 54), in the other group four-dimensional CT was the first-line imaging modality (*n* = 51). Sensitivity and positive predictive value were calculated on patient, lateralisation and localisation level. The need for additional imaging was also assessed for both groups.

**Results:**

Four-dimensional CT had a significantly higher sensitivity compared to the combination of ultrasound/[^99m^Tc]Tc-Sestamibi SPECT on patient and localisation level (70.6% vs. 51.9%, *p* = 0.049 and 60.8% vs. 35.2%, *p* = 0.009 respectively). Sensitivity for lateralisation also appeared higher, but did not reach significance (62.7% vs. 44.4%, *p* = 0.060). Positive predictive value was not significantly higher for four-dimensional CT compared to ultrasound and [^99m^Tc]Tc-Sestamibi SPECT (88.9% vs. 85.7% for lateralisation and 86.1% vs. 67.9% for localisation respectively). Additional imaging was required in 14 patients with four-dimensional CT as first-line imaging (27.4%) consisting of 2 ultrasound/[^99m^Tc]Tc-Sestamibi SPECT and 13 [^18^F]fluorocholine PET/CT, compared to 24 patients with ultrasound/[^99m^Tc]Tc-Sestamibi SPECT as first-line imaging (44.4%), requiring 22 four-dimensional CT and 9 [^18^F]fluorocholine PET/CT.

**Conclusions:**

Four-dimensional CT as the sole first-line parathyroid imaging modality had higher sensitivity than the combination of ultrasound and [^99m^Tc]Tc-Sestamibi SPECT, therefore requiring fewer additional procedures. Although the most costly, [^18^F]fluorocholine PET/CT was the most effective technique to localise parathyroid adenoma in case all other imaging was negative.

## Background

With a prevalence between 0.1 and 0.7%, primary hyperparathyroidism (PHPT) is a relative rare condition, yet the third most common endocrine disorder in adults following diabetes and thyroid diseases(Yu et al. 2009; Christensson et al. 1976; Wermers et al. 1997; MacKenzie-Feder et al. 2011; Fraser 2009; Yeh et al. 2013). PHPT is caused by an excess secretion of parathyroid hormone, resulting in hypercalcaemia. Single adenomas cause PHPT in 80% of patients, and multiglandular disease in the remainder (Felger and Kandil 2010; Erickson et al. 2022). Parathyroid carcinoma is a very rare cause of hyperparathyroidism, causing PHPT in less than 1% of all PHPT patients (Marx 2000). Adenomas are predominantly located dorsally to the superior or inferior pole of the thyroid gland. However, 15% of patients have ectopic parathyroid glands, the most common locations being mediastinal, intra-thymic, intrathyroidal or in the tracheooesophageal groove (Rosen et al. 2020; Noussios et al. 2012).

Originally PHPT was treated by surgical bilateral exploration of the neck without any prior imaging. With the introduction of minimally invasive parathyroidectomy, preoperative adenoma localisation has become essential. First-line imaging strategy in most hospitals is a combination of neck ultrasound (US) and [^99m^Tc]Tc-Sestamibi SPECT (MIBI). Sensitivity of this combination varies between 65% and 97.4% (Jong et al. 2021; Abd Elhameed Elsayed and Ali 2019). When the combination of US and MIBI SPECT has a negative or discordant result, further imaging is required. Depending on local experience and availability, this may be a four-dimensional computed tomography (4DCT) or [^18^F]fluorocholine PET/CT (FCH) with sensitivities ranging between 75 and 97% for 4DCT and 85-97% for PET/CT (Bahl et al. 2014; Bunch et al. 2022; Ballester Vazquez et al. 2020; Broos et al. 2019a, b). Few articles describe the role of 4DCT as first-line imaging technique (Jong et al. 2021; Leong et al. 2021). The present paper compares 4DCT as first-line imaging to the conventional combination of US and MIBI.

## Methods

### Patient selection

A database was created using the Picture Archiving and Communication System (PACS), searching for modalities of parathyroid imaging (US, MIBI, 4DCT, FCH-PET/CT) performed between January 2012 and January 2021 and using the Electronic Patient Record (EPR) system searching for surgery reports between January 2012 and January 2021 in our hospital. A total of 227 patients were identified and included in the database. Demographic information, laboratory values, comorbidities, medication, all preoperative imaging procedures (the order and the results) and the pathology report of the surgical specimen were recorded. The result were scored dichotomously as either positive or negative. When a positive result was reported, the location was categorised in laterality and quadrant (left, right, both or ectopic). When the quadrant was not precisely reported, the radiology images were re-evaluated by an experienced radiologist and the scintigraphic imaging was re-evaluated by an experienced nuclear medicine physician. The gold standard was defined as the localisation of the parathyroid adenoma during surgery, confirmed by a significant decline of intraoperative PTH measurement and histopathological confirmation of a parathyroid adenoma. This study was approved by the local ethics review board (Reference number 2020 − 1540).

### Imaging

In this study, two imaging strategies for adenoma localisation were compared, the combination of MIBI and US versus 4DCT, respectively. Imaging was considered positive when both MIBI and US correctly localised the adenoma and considered negative when there was no localisation or discordant localisation, as another modality (4DCT and/or FCH-PET/CT) would be required to localise the adenoma, being current clinical practice in many hospitals. Therefore, sensitivity and positive predictive value (PPV) were calculated only for the combination of the two modalities.

For MIBI, dual-phase static imaging of the neck was performed (Energy window 140.5 ± 10 keV, matrix 256 × 256, LEHRS collimator, 5 min acquisition). The first phase was recorded 15 min after i.v. injection of 550–600 MBq Tc-99- Sestamibi (Curium Netherlands B.V., Petten, The Netherlands). Delayed imaging was performed at 90 min p.i. both planar and SPECT (Brightview, Philips Medical Systems, Best, The Netherlands). SPECT acquisition parameters are energy window of 140.5 and 120 ± 10 keV, matrix 128 × 128 and LEHRS collimator.

Ultrasound was performed by experienced radiologists or experienced ultrasound technicians, supervised by an experienced radiologist, using a Toshiba Aplio 500 or a Philips EPIQ elite, equipped with a linear 12 MHz probe. US and MIBI were always performed within a two week time window, yet not reported by the same physician.

Using iodine-based intravenous contrast agents (Xenetix 300, Guerbet, Villepinte, France), 4DCT was acquired on a Philips Brilliance 40, Philips iCT 256 or Philips iQon, using four-phase scanning (pre-contrast, arterial, venous and late venous with post-threshold delays of 10, 40, and 85 s, respectively). Acquisition parameters were helical scan type, pitch 1.258, rotation time 0.27s, 120 kV, DoseRight index 14, collimation 64 × 0.625. Axial, coronal and sagittal reconstructions were made.

FCH-PET/CT was acquired on Philips ToF or Vereos PET/CT systems after administration of 210 MBq FCH. After 40 min PET/CT was performed, starting with a low-dose CT scan for attenuation correction and anatomical reference, followed by 20 min of PET acquisition.

Historically, the combination of MIBI and US was the first-line imaging procedure of choice. However, with increasing evidence on clinical utility, first-line use of 4DCT gradually increased in our hospital at the request of the referring endocrinologist. This organically created the two groups analysed in this study.

### Statistics

Sensitivity was calculated for each modality on a patient level, lateralisation level and localisation level. The patient level was defined as a positive result disregarding the location; lateralisation level was defined as the identification of the correct side and localisation level was defined as the identification of the correct quadrant. PPV was calculated in lateralisation and localisation analysis. Specificity and NPV were not calculated because of the impossibility of true negatives in this retrospective design. Chi-square analysis was used to determine a significant difference in sensitivity and PPV between the two groups. Furthermore, an independent t-test was performed to determine differences in laboratory values and length and weight of the adenoma between the patients in the two groups. A p-value of below 0.05 was considered statistically significant.

## Results

### Patients’ characteristics and selection

**Fig. 1:**
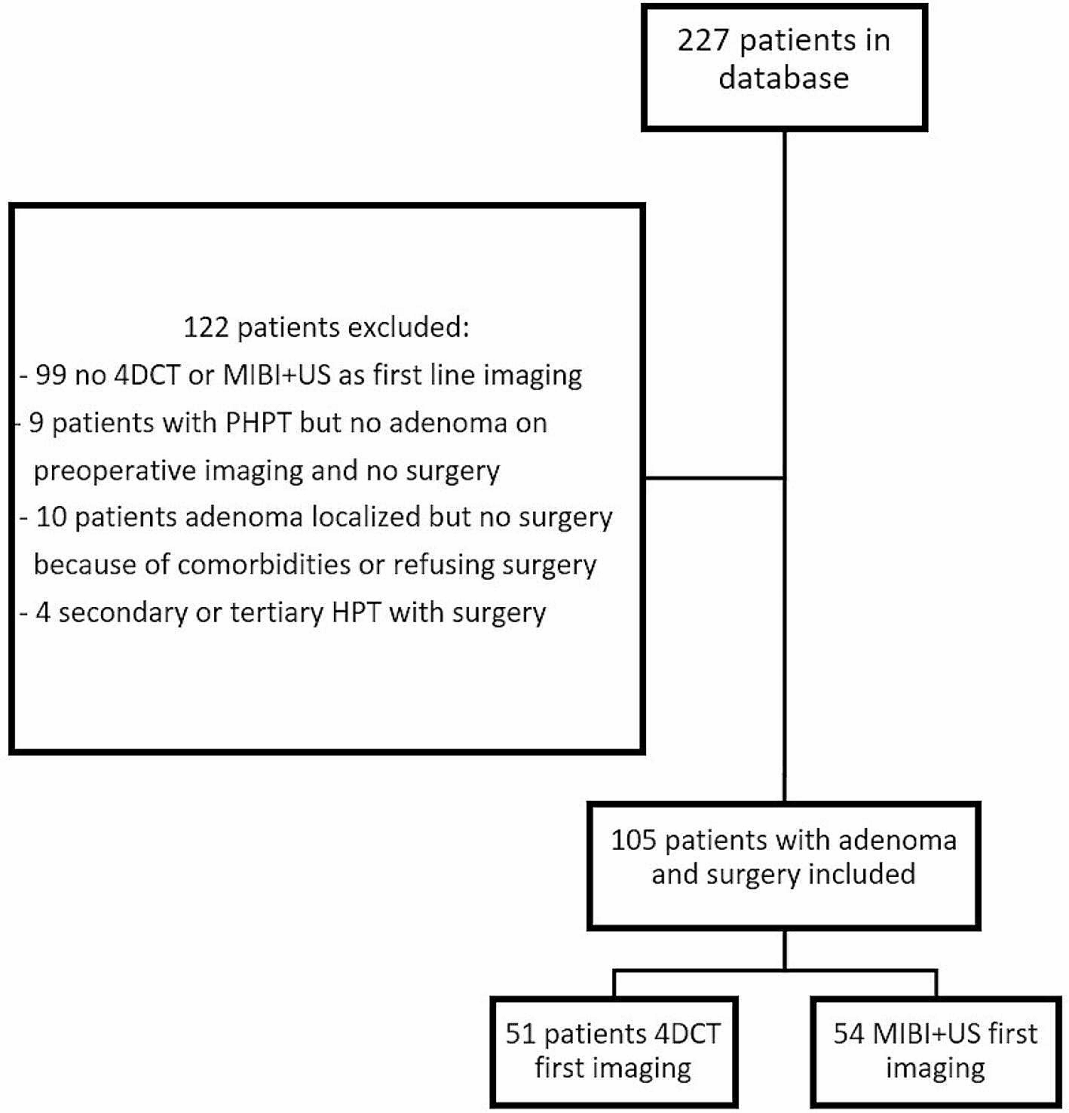
Inclusion of patients in the analysis

A total of 227 patients were included in the database using the PACS and EPR queries. Of these 227 patients, 122 patients were excluded for a variety of reasons, as shown in Fig. 1.

**Fig. 2:**
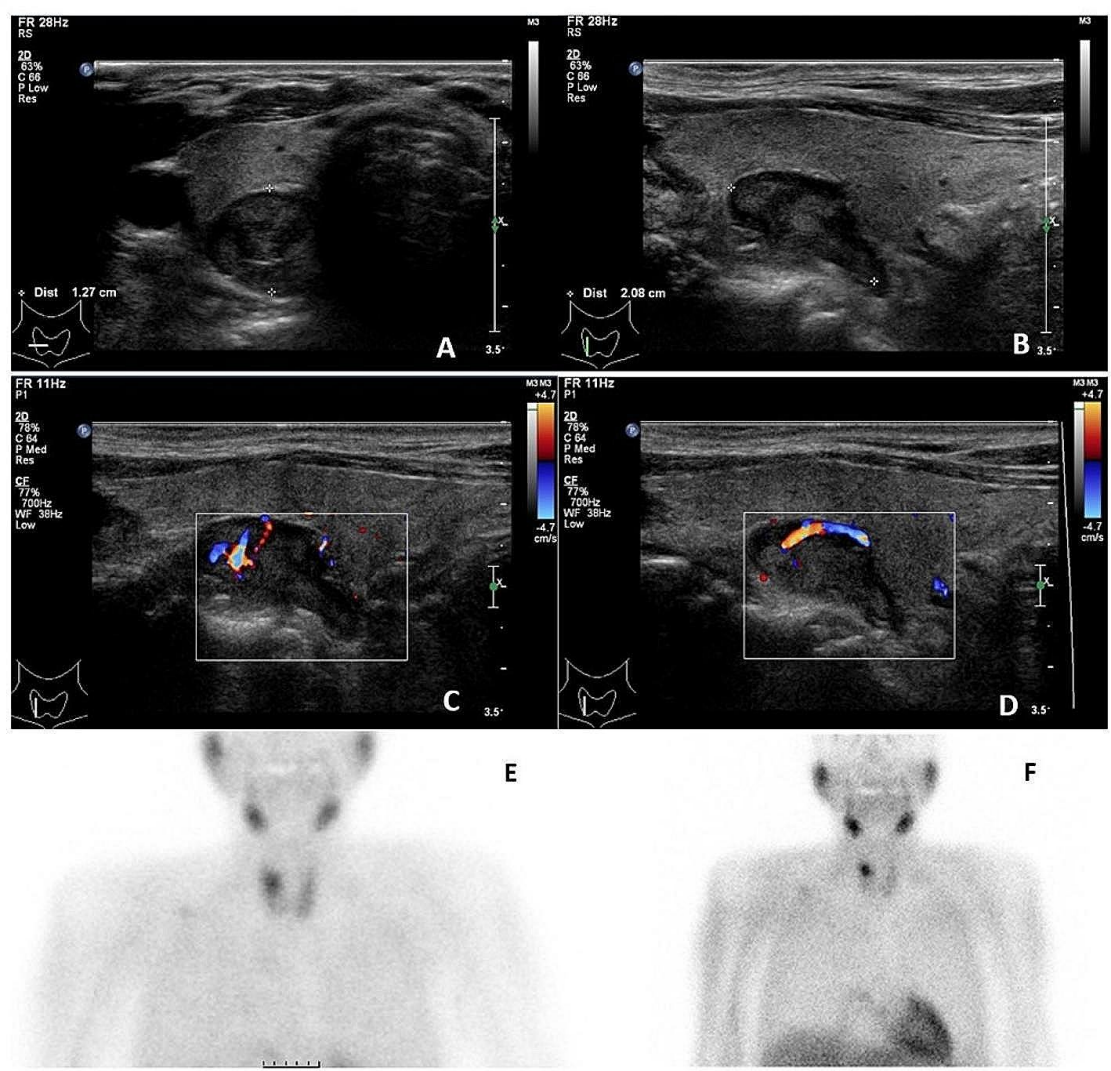
75-year-old male with asymptomatic primary hyperparathyroidism. US showed a hypoechoic lesion dorsally of the right upper lobe of the thyroid in transversal (**A**) and longitudinal (**B**) direction with characteristic polar vessel sign (**C**, **D**). MIBI confirmed Tc-99-Sestamibi uptake in the lesion after 15 min (**E**) with delayed wash-out compared to the thyroid gland at 90 min (**F**). The lesion was surgically removed and confirmed by histopathology to be a parathyroid adenoma

**Fig. 3:**
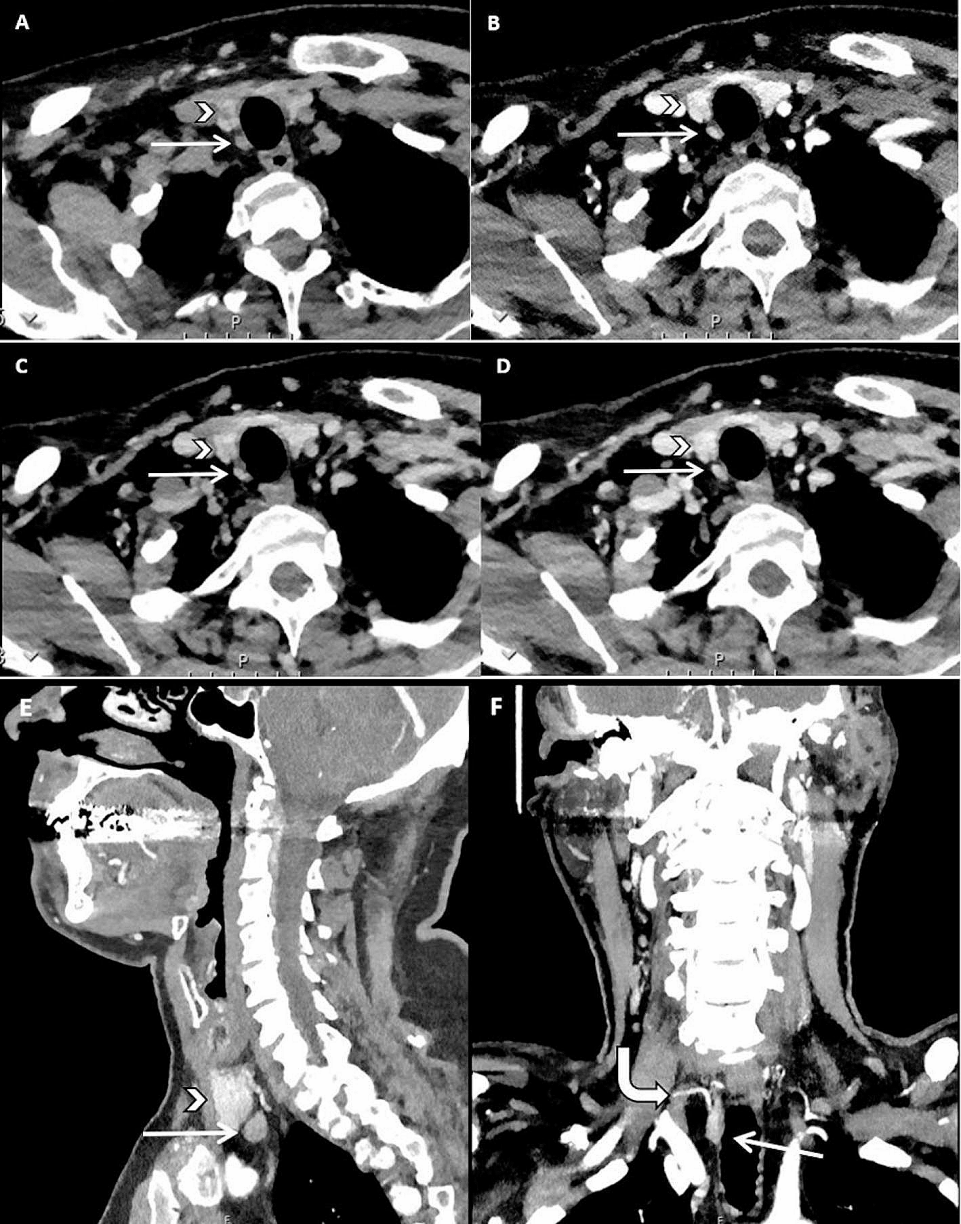
62-year-old male with asymptomatic primary hyperparathyroidism. 4DCT showed a hypodense lesion (white straight arrow) dorsally from the right lower quadrant of the thyroid (white arrowhead) on the non-enhanced image (**A**) with high arterial enhancement (**B**) and higher washout compared to the thyroid gland on the venous (**C**) and delayed venous (**D**) phase. Sagittal reconstruction showed the location of the lesion compared to the thyroid (**E**). Coronal maximum intensity projection (MIP) showed an enlarged artery (curved white arrow) feeding the lesion, also known as the polar vessel sign (**F**). The lesion was surgically removed and confirmed by histopathology to be a parathyroid adenoma

Hundred and five patients were included for further analysis. They either had a combination of US and MIBI or a 4DCT as first-line imaging, and all patients underwent surgery and had at least one parathyroid adenoma removed. The patient baseline characteristics of both first-line imaging approaches are shown in Table 1. Fifty-four patients had the conventional combination of US and MIBI as the first-line imaging modality and 51 patients had a 4DCT as first imaging modality. Figure 2 shows an example of a patient with the combination of US and MIBI as first-line imaging, Fig. 3 shows an example of a patient with 4DCT as first imaging modality. There were no significant differences between the two groups with respect to age, gender, serum total calcium or PTH.

**Table 1:** Patient characteristics of included patients per group, mean and range

Patient characteristics (mean)	*4DCT (n = 51)*	*MIBI + US (n = 54)*	P-value
Age (years)	58 (25–85)	63 (29–79)	0.053
Female gender	44 (86.3%)	43 (79.6%)	0.367
Preoperative total calcium (mmol/L)	2.89 (2.57–3.77)	2.98 (2.63–4.62)	0.147
Preoperative PTH (pmol/L)	20.4 (5.2–75.2)	22.4 (2.9–249.0)	0.723
Adenoma weight (grams)	1.7 (0.1–15.0)	2.1 (0.2–15.0)	0.591
Adenoma length (mm)	19 (2–45)	21 (10–60)	0.403

Out of 105 patients, 102 patients had a single adenoma (97.1%), of which five adenomas were in an ectopic location (4.8%). Three patients had two adenomas (2.9%).

No significant difference was found between the two groups in age, gender, weight or length of the adenoma or laboratory values like PTH or calcium. The outcome of any of the modalities could not be predicted by the examined parameters using a logistic regression model.

### Sensitivity & positive predictive value

Sensitivity of the imaging techniques is summarised in Table 2. Sensitivity was significantly higher for 4DCT on a patient level as compared to the conventional MIBI and US combination (70.6% and 51.8%, respectively, *p* = 0.049). Sensitivity for the correct side (lateralisation) was 62.7% for 4DCT and 44.0% for MIBI and US (not significantly different). The correct quadrant sensitivity was significantly higher for 4DCT compared to MIBI and US (60.8% and 35.2%, respectively, *p* = 0.009).

The experience of the head and neck radiologist seemed to influence sensitivity for 4DCT. Sensitivity increased from 70.6 to 76.5% on a patient level, from 62.7 to 66.7% for lateralisation and from 60.8 to 64.7% for correct quadrant localisation for a less experienced radiologist vs. an experienced head and neck radiologist.

**Table 2:** Sensitivity of first imaging modality in the two groups divided on patient, lateralisation and localisation level

Sensitivity	4DCT	MIBI + US	P-value
Patient	70.6%	51.9%	0.049*
Lateralisation	62.7%	44.4%	0.060
Localisation	60.8%	35.2%	0.009*

*=Significant difference, p-value < 0.05

As shown in Table 3, the positive predictive value was similar for identification of the correct side in both groups, 88.9% for 4DCT and 85.7% for MIBI and US. The difference in positive predictive value for the correct quadrant between 4DCT (86.1%) and MIBI / US (67.9%) did not reach statistical significance.

**Table 3:** Positive predictive value (PPV) of the two groups divided on lateralisation and localisation level

PPV	4DCT	MIBI + US	P-value
Lateralisation	88.9%	85.7%	0.703
Localisation	86.1%	67.9%	0.080

### Additional imaging

Additional imaging was required for patients in both groups to localise the parathyroid adenoma in case of negative first-line imaging, as shown in Table 4. In the group with 4DCT as first-line imaging and a negative result, 13 FCH-PET/CTs were acquired. 11/13 were positive for an adenoma on FCH-PET/CT (84%). Only 2 MIBI / US were ordered for patients with negative 4DCT results, one of the two was positive for the localisation of the adenoma.

In the group with MIBI and US as the first-line of imaging, a total of 22 4DCTs were acquired when MIBI / US had a negative or discordant result. A total of 16 4DCTs showed a positive localisation for the adenoma (72.7%), and 6 were negative. FCH-PET/CT was performed in 9 patients with negative or discordant MIBI and US results, all of which were successful in localising the adenoma, including the 6 patients with negative 4DCT outcome.

**Table 4:** Additional imaging required in the two groups per modality

Additional imaging	*4DCT (n = 51)*	*MIBI + US (n = 54)*
MIBI	2 (3.9%)	-
US	2 (3.9%)	-
4DCT	-	22 (40.7%)
FCH-PET/CT	13 (25.5%)	9 (16.7%)

Overall sensitivity of FCH-PET/CT in this study population was 90.9% (20 out of 22 patients).

**Fig. 4:**
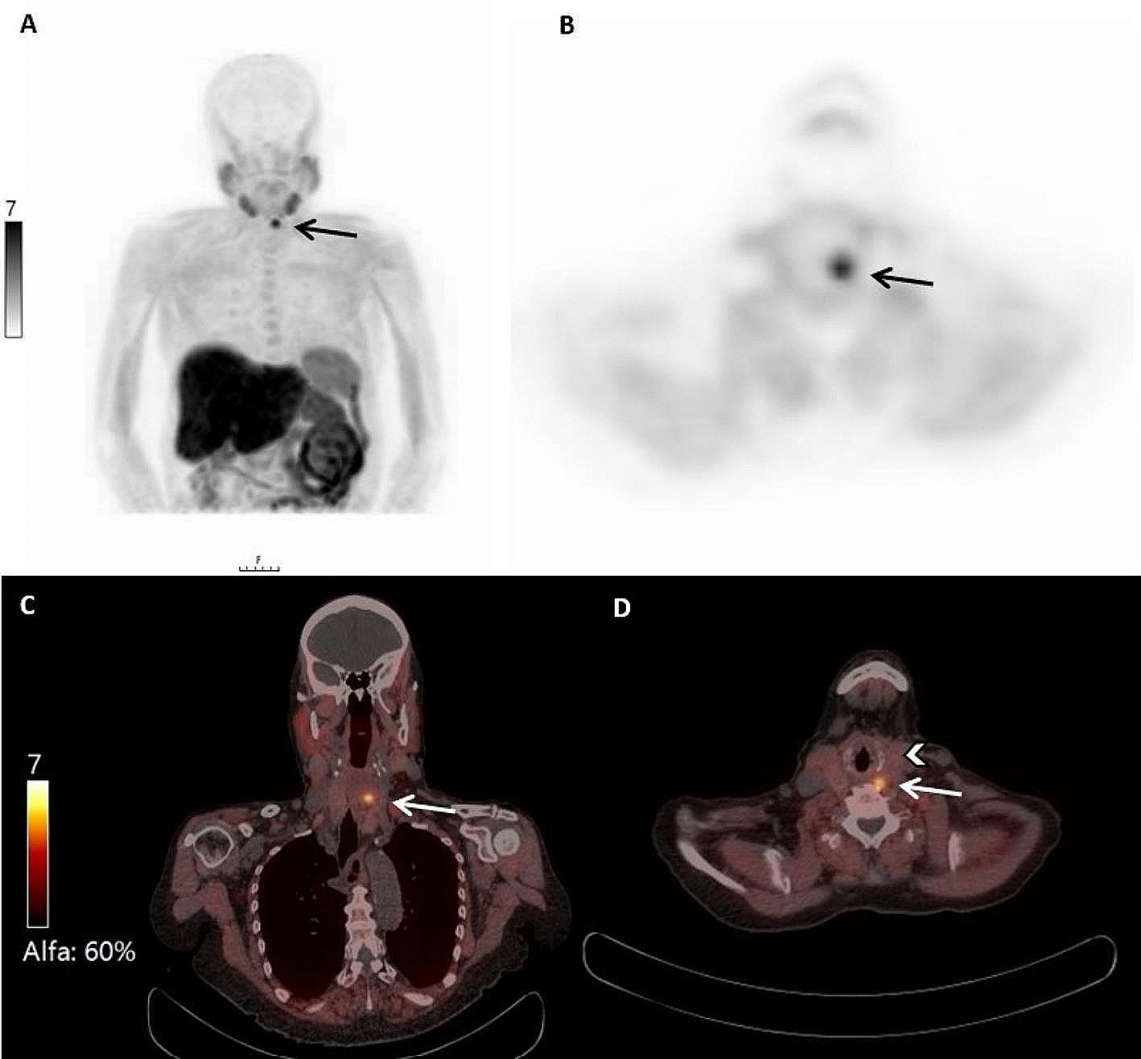
73-year-old female with asymptomatic primary hyperparathyroidism. After negative US, MIBI and 4DCT, a FCH-PET-CT was performed. Coronal MIP (**A**) and transverse PET image (**B**) show FCH uptake in a lesion (black arrow) on the left upper quadrant of the thyroid gland. Fusion images in coronal (**C**) and transverse (**D**) direction show the position of the lesion (white arrow) dorsally of the thyroid gland (white arrowhead). The lesion was surgically removed and confirmed by histopathology to be a parathyroid adenoma

See Fig. 4 for an example of FCH-PET/CT after negative preoperative imaging.

The group with 4DCT as first-line imaging required fewer additional imaging compared to the group of MIBI and US as first-line imaging.

## Discussion

The aim of our study was to evaluate the use of 4DCT as a first-line imaging strategy compared to the combination of US and MIBI. First-line 4DCT resulted in fewer additional imaging and had higher sensitivity. Performing only 4DCT as first-line imaging would likely be a more time and cost-efficient strategy to localise the adenoma.

The higher sensitivity of 4DCT compared to US and MIBI is in line with results from previous studies. De Jong et al. showed that the combination of US and 4DCT had the highest sensitivity as a first-line imaging strategy, higher than the combination of US and MIBI (88% vs. 65%). 4DCT alone has a sensitivity of 87%, using a three-phase protocol (Jong et al. 2021). Leong et al. also showed a high sensitivity by using 4DCT as first-line imaging, with a sensitivity of 82.5%, which was higher for head and neck radiologists compared to general radiologists (Leong et al. 2021). Vu et al. showed the highest accuracy for quadrant localisation for the combination of 4DCT and MIBI (93.5%) followed by 4DCT alone (87.1%) and MIBI alone (67.7%) (Vu et al. 2019).

One of the drawbacks of 4DCT in literature is the higher radiation dose compared to the combination of MIBI and US. The radiation dose is highly dependent on the scan protocol. In literature reported effective radiation dose for 4DCT ranges from 5.2 to 28 mSv and MIBI SPECT dose ranges between 3.33 and 12 mSv (Mahajan et al. 2012; Hoang et al. 2015; Campbell et al. 2015; Yeh et al. 2019; Starker et al. 2011; Madorin et al. 2013). The average dose length product in our study was 536 mGy*cm, with a calculated effective dose of 6.1 mSv for our 4DCT protocol, compared to 5.4 mSv for our MIBI SPECT protocol.

One way to reduce radiation exposure from 4DCT is by reducing the number of phases. Our protocol for 4DCT consists of four phases, however, different combinations have been investigated in literature with variable results. Raghavan et al. showed a lateralisation and localisation accuracy of 90.5% and 91.5% respectively for the arterial phase alone (Raghavan et al. 2014). Griffith et al. studied a two-phase CT protocol consisting of an arterial and venous phase with a sensitivity for lateralisation of 78.8% and a localisation sensitivity of only 55.4% (Griffith et al. 2015). Seyednejad et al. used a two-phase protocol consisting of a non-enhanced and dual-energy venous phase scan, resulting in a sensitivity of 90% (Seyednejad et al. 2016). Further research regarding the optimal combination of phases is still needed.

Several studies demonstrated the utility of FCH-PET/CT as a first-line imaging technique. Broos et al. reported a detection rate of 90–96% for FCH- PET/CT as a first-line imaging (Broos et al. 2019a, b). Hocevar et al. showed a sensitivity of 98% in 151 patients with a high positive predictive value of 96.2%. The operative success rate was more than 95% when only preoperative FCH-PET/CT was used as first-line imaging, without intraoperative PTH testing (Hocevar et al. 2017). Thanseer et al. compared US, MIBI and FCH-PET/CT as first-line imaging, with a sensitivity of 100% for PET/CT compared to 69.3% and 80.7% for US and MIBI respectively (Thanseer et al. 2017). Beheshti et al. compared FCH-PET/CT to MIBI or Tc-99 m-tetrofosmine SPECT/CT in 100 patients. They reported a sensitivity of FCH-PET/CT of 93.7% compared to 60.8% for SPECT/CT (Beheshti et al. 2018). Bossert et al. compared US, MIBI and FCH-PET/CT in 34 patients, with sensitivities of 68%, 15% and 71% respectively (Bossert et al. 2019). Most studies showed a high sensitivity for FCH-PET/CT, as also demonstrated in the current study in which FCH-PET/CT showed a high detection rate, even when all other imaging procedures failed. However, the relative cost of the radiopharmaceutical and availability of FCH-PET/CT hampers its wide-spread use as a first-line imaging (Broos et al. 2019a, b).

There are several limitations to our study. It is a retrospective study in a single institution and patient numbers are relatively small. A prospective study comparing all three imaging techniques as first-line imaging in a single group of patients would of course be more accurate. With increasing literature on 4DCT there was a natural shift in the type of first-line imaging used, however, because of the retrospective nature of the study, there was no randomisation of patients in the two categories of first-line imaging. Because of the relatively long period, there were also upgrades in the machines used for the US, MIBI and CT. Imaging results were used as initially reported, acknowledging a higher detection rate of parathyroid adenoma by expert review, thereby providing a conservative, real-world estimate of sensitivity. Sensitivity was higher when reporting was done by expert head and neck radiologists and nuclear medicine physicians as compared to less experienced head and neck radiologists and nuclear medicine physicians.

## Conclusion

4DCT as the sole first-line parathyroid imaging modality has a higher sensitivity than the combination of US and MIBI, therefore requiring fewer additional imaging procedures. Although the most expensive, FCH-PET/CT remains the most effective technique to localise parathyroid adenoma, also in case all other imaging is negative.

## Data Availability

The datasets used and/or analysed during the current study are available from the corresponding author upon reasonable request.

## Abbreviations

4DCT: four-dimensional computed tomography
EPR: Electronic Patient Records
FCH-PET/CT: [^18^F]fluorocholine PET/CT
MIBI: [^99m^Tc]Tc-Sestamibi SPECT
PACS: Picture Archiving and Communication System
PHPT: primary hyperparathyroidism
PPV: positive predictive value
PTH: parathyroid hormone
US: ultrasound
